# Emodin reduces surgical wounding‐accelerated tumor growth and metastasis via macrophage suppression in a murine triple‐negative breast cancer model

**DOI:** 10.14814/phy2.15813

**Published:** 2023-10-11

**Authors:** Sierra J. McDonald, Brooke M. Bullard, Brandon N. VanderVeen, Thomas D. Cardaci, Ioulia Chatzistamou, Daping Fan, E. Angela Murphy

**Affiliations:** ^1^ Department of Pathology, Microbiology & Immunology, School of Medicine University of South Carolina Columbia South Carolina USA; ^2^ Department of Cell Biology and Anatomy, School of Medicine University of South Carolina Columbia South Carolina USA; ^3^ AcePre, LLC Columbia South Carolina USA

**Keywords:** breast cancer, emodin, lung metastasis, macrophages, surgical wounding

## Abstract

It has been suspected that tumor resection surgery itself may accelerate breast cancer (BC) lung metastasis in some patients. Emodin, a natural anthraquinone found in the roots and rhizomes of various plants, exhibits anticancer activity. We examined the perioperative use of emodin in our established surgery wounding murine BC model. Emodin reduced primary BC tumor growth and metastasis in the lungs in both sham and surgical wounded mice, consistent with a reduction in proliferation and enhanced apoptosis (primary tumor and lungs). Further, emodin reduced systemic inflammation, most notably the number of monocytes in the peripheral blood and reduced pro‐tumoral M2 macrophages in the primary tumor and the lungs. Consistently, we show that emodin reduces gene expression of select macrophage markers and associated cytokines in the primary tumor and lungs of wounded mice. Overall, we demonstrate that emodin is beneficial in mitigating surgical wounding accelerated lung metastasis in a model of triple‐negative BC, which appears to be mediated, at least in part, by its actions on macrophages. These data support the development of emodin as a safe, low‐cost, and effective agent to be used perioperatively to alleviate the surgery triggered inflammatory response and consequential metastasis of BC to the lungs.

## Graphical abstract

We report that natural compound emodin reduced surgical wounding‐accelerated lung metastasis in a model of triple‐negative breast cancer (BC) by targeting macrophages. Our data support the development of emodin as a safe, low‐cost, and effective agent to be used perioperatively to alleviate the surgery triggered systemic inflammatory response and reduce resulting metastasis of BC. This is relevant as there is no approved preventive therapeutic for BC metastasis, especially for hormone receptor negative patients.

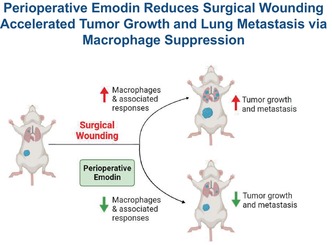

## 1 INTRODUCTION

Breast cancer (BC) is the second leading cause of cancer‐associated death in women in the United States (Siegel et al., 2022). Roughly, one‐third of all BC mortality can be attributed to metastatic recurrence, most notably in the lungs or bones, following preliminary success of surgery and/or other cancer therapeutics (Cheng et al., 2012; Colleoni et al., 2016; Jin et al., 2018). It has been suspected that tumor resection surgery may accelerate cancer metastasis in some patients (Baum et al., 1999; Demicheli et al., 2007; Fisher & Fisher, 1959; Retsky et al., 2008). Indeed, clinical evidence strongly favors the hypothesis that clinically untraceable cancer cells termed micrometastases are existent in distant organs preceding surgery but remain quiescent and under immune regulation, until this metastatic latency is disturbed by the surgical procedure itself to result in enhanced enlargement of micrometastases into clinically measurable macrometastases (Demicheli et al., 2007; Dillekas et al., 2014, 2016; Klein, 2020). Given that it is impractical and unethical to perform pseudo‐surgery to directly test this hypothesis in patients, our group and others have devised mouse models to link surgical wounding to the hastening of local as well as distant tumor development (i.e., lung metastasis) (Krall et al., 2018; McDonald, VanderVeen, Bullard, et al., 2022). We recently reported that the surgical wounding procedure, at a remote site and in the absence of tumor resection, is adequate to enhance primary tumor succession and promotion of lung metastasis in a murine model of triple‐negative breast cancer (TNBC) (McDonald, VanderVeen, Bullard, et al., 2022). These findings were accompanied by an upsurge in M2 macrophages, mediators of pro‐tumoral processes, in the primary tumor as well as in the lungs of wounded mice (McDonald, VanderVeen, Bullard, et al., 2022) that is assumed to be driven by the general inflammatory response generated by the wounding process.

Currently, systemic adjuvant therapies are administered to prevent metastasis to the lungs and other sites; however, these therapies are only effective in around 20% of BC patients, with the majority exhibiting a recurrence several months or years after (Cole et al., 2001; Gianni et al., 2011). Despite the effectiveness of aromatase inhibitors and selective estrogen receptor (ER) modulators in averting metastatic recurrence of BC, their benefits are limited to ER+ BC and their side effects make them less than desirable for long‐term use (Patel & Bihani, 2018). Presently, there are no agents for the prevention of ER‐ BC. This is of significance due to the elevated metastatic recurrence rate, most frequently in the lungs, and the consequential poor prognosis of TNBC (Waks & Winer, 2019). Emodin (1,3,8‐trihydroxy‐6‐methylanthraquinone), derived from the roots and barks of select plants, is a Chinese small molecule compound that has exhibited antitumor potential in preclinical models, including models of breast cancer and lung cancer (Iwanowycz, Wang, Hodge, et al., 2016; McDonald, VanderVeen, Velazquez, et al., 2022). Our labs discovered that emodin inhibits BC growth and lung metastasis in orthotopic murine models by diminishing macrophage recruitment to tumors and lungs and suppressing their polarization to tumor‐associated macrophages (Iwanowycz, Wang, Altomare, et al., 2016; Iwanowycz, Wang, Hodge, et al., 2016). However, its potential in reducing surgical wounding‐accelerated BC tumor growth and lung metastasis has not yet been examined.

We hypothesize that emodin can be developed as an effective complementary agent to be used perioperatively to alleviate the surgery‐triggered systemic inflammatory response and reduce resulting BC lung metastasis. We used our previously established model of surgical wounding‐accelerated BC growth and lung metastasis to examine the ability of emodin to reduce BC metastasis to the lungs following surgical wounding (McDonald, VanderVeen, Bullard, et al., 2022). We demonstrate that emodin significantly reduces primary tumor growth and M2 pro‐tumor macrophages in both BC wounded and BC sham mice in a TNBC model. Importantly, we report that emodin ameliorates the surgical wounding‐accelerated lung metastasis in BC. These data support the further advancement of emodin as a safe, low‐cost, and effective agent for reducing primary tumor growth and mitigating surgical wounding‐accelerated lung metastasis in TNBC.

## 2 MATERIALS AND METHODS

### 2.1 Animals

Five‐week‐old female BALB/c (*n* = 50) mice were purchased from Jackson laboratories and cared for in the Department of Laboratory Animal Resources (DLAR) at the University of South Carolina's School of Medicine. Mice were housed 3–5/cage, maintained on a 12:12‐h light–dark cycle in a low‐stress environment (22°C, 50% humidity, low noise), and given food and water ad libitum. All mice were fed an AIN‐76A diet (Bioserv, catalog#:F1515), a purified, balanced diet that is phytoestrogen free, (Enos et al., 2013; Grotto & Zied, 2010) upon arrival (5 weeks of age) and through the study duration (until 15 weeks of age). Dietary phytoestrogens have been shown to influence anxiety‐related behaviors, fat deposition, blood insulin, leptin and thyroid levels as well as lipogenesis and lipolysis in adipocytes, all of which could nonspecifically impact tumorigenesis (Warden & Fisler, 2008). All experimental procedures started at 10 weeks of age. Body weight, food, and water consumption were monitored on a weekly basis for the duration of the study (5 weeks). All experimental mice were euthanized 5 weeks post BC cell inoculation (15 weeks of age). The Institutional Animal Care and Usage Committee of the University of South Carolina approved all experiments, and all methods were performed in accordance with the American Association for Laboratory Animal Science.

### 2.2 Experimental design

**FIGURE 1:**
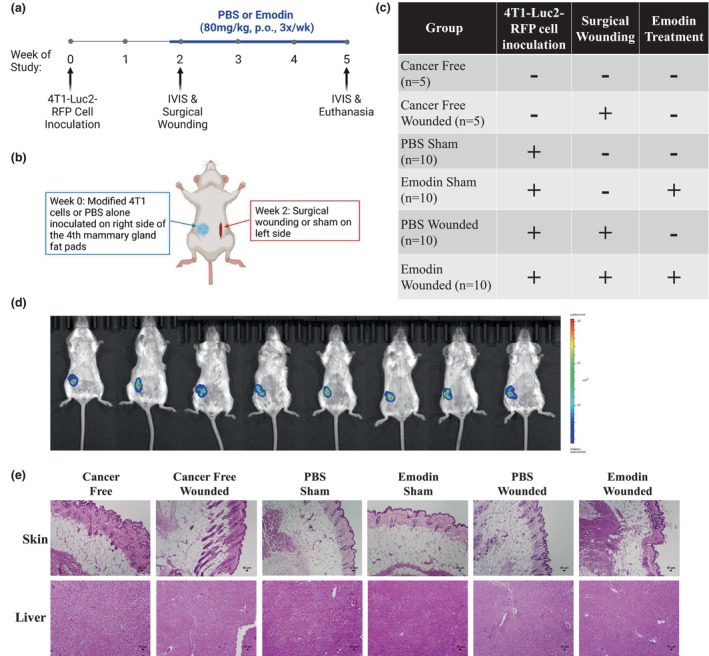
Experimental design and animal characteristics. (a) Experimental timeline for 10‐week‐old female BALB/c mice inoculated with either PBS (*n* = 10) or 1 × 10^4^ 4 T1‐Luc2‐RFP cells (*n* = 40) at week 0. Oral administration of PBS (vehicle; *n* = 30) or emodin (*n* = 20) was performed 2 days prior to wounding and three times a week thereafter. Schematic created using BioRender. (b) Schematic for all mice inoculated with either PBS or 4T1‐Luc2‐RFP cells on the right side of the fourth pair mammary gland fat pads at Week 0. Two weeks postinoculation, mice were either subjected to a 2‐cm long cutaneous incision (wounded) or no incision (sham) on the contralateral side of the tumor. Schematic created using BioRender. (c) List of groups with appropriate experimental characteristics including tumor cell inoculation, surgical wounding, and emodin treatment. (d) Representative bioluminescent images of luciferase carried 4 T1‐Luc2‐RFP tumor cells in all mice before surgical wounding using in vivo IVIS at Week 2. (e) Representative H&E‐stained section of skin (10x) on left side of mouse and liver (10×) at 5 weeks post 4T1‐Luc2‐RFP cell inoculation. Scale bar = 50 μm.

We took advantage of our novel generation of a TNBC cell line, 4T1‐Luc2‐RFP, that displays delayed breast tumor progression and reduced lung metastatic occurrence in comparison with the rapid growth and metastatic occurrence of parental 4T1 breast tumors (Atiya et al., 2019). We utilized our previously published model for surgical wounding (McDonald, VanderVeen, Bullard, et al., 2022). Briefly, 10‐week‐old female BALB/c mice were inoculated with 1 × 10^4^ 4T1‐Luc2‐RFP cells (*n* = 40) on the right side of the fourth pair mammary gland fat pads at Week 0, PBS was used as control (*n* = 10) (Figure 1a,b). Once tumors were established, reaching approximately 200 mm^3^ in size 2 weeks postinoculation, mice with similar tumor sizes (determined by IVIS imaging) were randomized to one of four groups (Figure 1c,d), and either subjected to a sterile 2 cm long cutaneous incision (wounded; *n* = 20) or no incision (sham; *n* = 20) on the contralateral side of the tumor. The wound was subsequently closed using one staple. Thus, a decoupled wounding model was used: an orthotopic tumor inoculated on the right side and a surgical wound created on the left side of a mouse. To model surgery wound healing, the incision was allowed to heal (Figure 1e).

### 2.3 Emodin administration

Vehicle or emodin was administered three times per week to mice from 2 days prior to sham or surgical wounding until the completion of the study (i.e., ~12 weeks until 15 weeks of age). Thus, a total of six groups were used: cancer free (*n* = 5), cancer‐free wounded (*n* = 5), PBS sham (*n* = 10), emodin sham (*n* = 10), PBS wounded (*n* = 10), and emodin wounded (*n* = 10) (Figure 1c). Emodin was purchased from Nanjing Langze Medicine and Technology Co. Ltd. and dissolved in DMSO as stock. It was subsequently diluted in phosphate‐buffered saline (PBS) (VWR; catalog# MRGF‐6235‐010Q) with final 1% DMSO and administered to mice P.O. at a dose of 80 mg/kg (24 mg/mL). PBS (with 1% DMSO) was used for vehicle control.

### 2.4 Tumor palpations

Tumors were measured beginning at 2 days following the inoculation by the same investigator. Mice develop a palpable mammary tumor 1 week post BC cell inoculation. Upon palpation of a tumor, calipers were used to measure the longest and shortest diameter of the tumor. Tumor volume was estimated using the formula: 0.52 × (largest diameter) × (smallest diameter)^2^ as previously described (Cranford et al., 2019; Steiner et al., 2014).

### 2.5 In vivo measurement of breast tumor growth and lung metastasis by IVIS imaging

Bioluminescent imaging was performed before wounding (2 weeks post tumor inoculation at 12 weeks of age) and before euthanasia (3 weeks post wounding at ~15 weeks of age) using a highly sensitive, IVIS Spectrum in vivo imaging system (PerkinElmer). D‐luciferin (Gold Biotechnology) was prepared per manufacturer's instructions in Dulbecco's phosphate‐buffered saline (DPBS) and filter sterilized with a 0.2‐μm filter for a concentration of 15 mg/mL. For all in vivo imaging, D‐luciferin solution was intraperitoneally injected (150 mg/kg) in all mice. Mice were then anesthetized with 2% isoflurane prior and during imaging with IVIS spectrum. Bioluminescence of D‐luciferin carried 4T1‐Luc2‐RFP tumor cells was quantified using Living Image, in which regions of interest from displayed images were identified and quantified as the total flux which is the radiance (photons/sec) in each pixel summed or integrated over the ROI area.

### 2.6 Tissue collection

Five weeks post BC cell inoculation, mice were euthanized by isoflurane overdose following a 4 h fast. Blood was collected from the inferior vena cava for a complete blood panel analysis. Breast tumors were excised, weighed (g), and measured (mm^3^) prior to cutting into fourths. One‐fourth portion of breast tumor was placed in RPMI 1640 medium (ATCC) supplemented with 10% FBS, 1% Penicillin/Streptomycin on ice for flow cytometry. Another one‐fourth portion of breast tumor was fixed in 10% neutral‐buffered formalin and paraffin embedded for histopathology, immunohistochemistry, and immunofluorescence. The remaining portions were snap frozen in liquid nitrogen for RT‐PCR gene expression analysis. A portion of the cutaneous skin was then carefully removed on the contralateral side of the tumor where mice were either subjected to a 2 cm incision (wounded) or no incision (sham). The skin tissue was fixed in 10% neutral‐buffered formalin and paraffin embedded for histopathology. Lungs and spleen were then excised and weighed. After which, lungs were imaged on IVIS as described below, and then fixed in 10% neutral buffered formalin and paraffin embedded for histopathology, immunohistochemistry, and immunofluorescence. Liver was fixed in 10% neutral‐buffered formalin and paraffin embedded for histopathology.

### 2.7 Blood panel analysis

A complete blood panel analysis was performed using the VetScan HMT (Abaxis) for determination of white blood cells (WBC), lymphocytes (LYM), monocytes (MON), neutrophils (NEU), and platelets (PLT). The neutrophil to lymphocyte ratio (NLR) was calculated from obtained values. Briefly, whole blood was placed in an EDTA coated microtube and analyzed on the VetScan HMT according to the manufacturer's instructions.

### 2.8 Ex vivo measurement of lung metastasis by IVIS imaging

For ex vivo imaging, lungs were carefully washed in a well containing PBS, and then transferred to another well in which D‐luciferin (300 μg/mL) was added to cover the tissue. Ex vivo lungs were immediately imaged on IVIS to determine lung nodule counts. Bioluminescence of D‐luciferin carried 4T1‐Luc2‐RFP tumor cells was quantified as the total flux as described above.

### 2.9 Flow cytometry

One‐fourth portion of breast tumors was cut into small fragments with a blade and enzymatically digested in 1 mL of RPMI 1640 medium (ATCC) supplemented with 10% FBS, 1% Penicillin/Streptomycin, 0.3 mg/mL of collagenase Type 4 (Worthington), 200 U/mL of DNase I (Worthington), and 1 U/mL of hyaluronidase (Sigma‐Aldrich) for 60 min at 37°C. After digestion, cell suspensions were passed through 70‐μm cell strainers. For the staining of cell surface markers (Biolegend unless otherwise indicated), appropriate samples were stained with ZombieGreen (FITC; Bio‐Rad) solution for 20 min at RT in dark and then washed with flow buffer (0.5% FBS, 10 mM HEPES (Gibco), and 2 mM EDTA (Gibco) in HBSS without calcium or magnesium). Cells were incubated with FC Block solution at 4°C for 10 min and then incubated with fluorescently labeled antibodies against CD45 (PE/Cy7), CD11b (APC), CD68 (APC/Cy7), CD206 (PE), and CD11c (PerCP/Cy5.5) at 4°C for 20 min, followed by two PBS washes with the final resuspension in flow buffer. Cells were measured using a FACS Aria II (BD) and analyzed using FlowJo (BD Biosciences).

Breast tumor cells were gated for non‐debris singlets and considered live immune cells with ZombieGreen^Neg/Low^ and CD45^+^. From the Live CD45^+^ population, CD11b^+^CD68^+^ cells were identified as macrophages. From the CD11b^+^CD68^+^ macrophage population, macrophage phenotype was determined based on CD206 and CD11c expression. CD206^−^CD11c^−^ cells were identified as M0 macrophages; CD206^−^CD11c^+^ as M1 macrophages; CD206^+^CD11c^−^ cells as M2 macrophages; CD206^+^CD11c^+^ as M1‐M2 transitional macrophages.

### 2.10 Real‐time quantitative PCR

Mammary tumors and lungs were homogenized, and RNA was extracted using the RNeasy Lipid Tissue Mini Kit (Qiagen, Cat#74804) according to the manufacturer's instructions. RNA sample quality and quantities were verified using a Nanodrop One Microvolume UV‐Vis Spectrophotometer (Thermo Fisher Scientific) and determined to be of good quality based on A260/A280 and 260/230 values (>1.8) prior to cDNA synthesis using High‐capacity Reverse Transcriptase kit (Applied Biosystems, Cat#4368814). Quantitative reverse transcriptase polymerase chain reaction (PCR) analysis was carried out as per the manufacturer's instructions and all primers used were TaqMan Gene Expression Assays (Applied Biosystems). TaqMan reverse transcription reagents were used to reverse transcribe RNA to cDNA in the mammary tumors (*n* = 5/group) and in the lungs (*n* = 3‐4/group). Gene expression of the following markers were assessed in the mammary tissue as previously described (Enos et al., 2015): monocyte/macrophage (EMR1/F480 and CD68), pro‐inflammatory M1 macrophage markers (IFNγ, IL‐1β, IL‐6, and TNF), anti‐inflammatory, and pro‐tumoral M2 macrophage markers (IL‐4, MSR1, and TGF‐β1), angiogenesis (VEGF), T cells (CD4 and CD8), neutrophils (Ly6G), and antiapoptotic Bcl‐2 in the mammary tissue. Additionally, gene expression of the following markers were assessed in the lungs: monocyte/macrophage (EMR1/F480 and CD68), pro‐inflammatory M1 macrophage markers (IL‐1β and IL‐6), anti‐inflammatory, and pro‐tumoral M2 macrophage markers (IL‐4 and TGF‐β1). Briefly, quantitative RT‐PCR analysis was carried out as per the manufacturer's instructions (Applied Biosystems) using Taq‐Man Gene Expression Assays on a Qiagen Rotor‐Gene Q. Data were normalized to control (PBS wounded mice) and compared to five reference targets (Hmbs, B2M, TBP, H2afv, and 18s), which were evaluated for expression stability using GeNorm.

### 2.11 Immunohistochemistry

To examine cell proliferation, immunohistochemical analysis of Ki67 (Abcam, #ab16667) was performed according to manufacturer's instructions (abcam IHC staining protocol for paraffin sections) with minor modifications to our previously described work (McDonald, VanderVeen, Bullard, et al., 2022). Briefly, color was developed with DAB (Vector Laboratories, #SK‐4100) as a chromogen for 6 min at RT followed by counterstaining using CAT hematoxylin and Tacha's bluing for 30 secs. Sections were dehydrated and mounted with Permount medium (Fisher Chemical, #SP15‐100). Representative images were taken at ×20 using Keyence All‐in‐One fluorescence Microscope BZ‐X800. Percentage of Ki67 cells (DAB‐positive nuclei) over total cells, relative to area, were quantified using Material Image Processing and Automated Reconstruction (MIPAR) software, more specifically, we used MIPAR's immunohistochemistry recipe which quantifies positively stained DAB cells relative to area (Rizzardi et al., 2012). The average of positively stained DAB cells relative to area from five images were assessed for each mouse (*n* = 3 mice/group). TUNEL staining was carried out as per the manufacturer's instructions using ApopTag Peroxidase In Situ Apoptosis Detection Kit (Millipore, #S7100). Color was developed with DAB (Vector Laboratories, #SK‐4100) as a chromogen for 6 min at RT followed by counterstaining using 1% methyl green (R&D systems, #4800‐30‐18) for 10 min at RT and then washed with diH20 and 100% N‐Butanol (Acros‐Organic, #71–36‐3). Sections were dehydrated and mounted with Permount medium (Fisher Chemical, #SP15‐100). Representative images were taken at ×20 using Keyence All‐in‐One fluorescence Microscope BZ‐X800. Percentage of apoptotic cells (TUNEL‐positive nuclei) over total cells, relative to area, were quantified using Material Image Processing and Automated Reconstruction (MIPAR) software, more specifically, we used MIPAR's immunohistochemistry recipe which quantifies positively stained DAB cells relative to area (Rizzardi et al., 2012). Five images were assessed for each mouse (*n* = 3 mice/group).

### 2.12 Immunofluorescence

After deparaffinization and rehydration, paraffin sections were incubated with rodent decloaker (Biocare Medical, #RD913M) at 95°C for 30 min for antigen retrieval and then washed with TBS. To block nonspecific binding, sections were incubated with 10% goat serum in 5% BSA/TBS for 1 h at RT in a humid chamber under gentle agitation. Following the removal of the block buffer, sections were labeled with primary antibody against CD206 (1:100, Novus Biologicals, Cat#90020) at 4°C overnight, washed with TBS, and then detected with Alexa Fluor 594 secondary antibody (1:200, Abcam, ab150080) in 5% BSA/TBS for 1 h at RT in a humid chamber under gentle agitation in the dark. Following secondary antibody, sections were washed with TBS and labeled with FITC conjugated F4/80 (1:150, Bio Rad, MCA497FB) for 4 h at RT in a humid chamber. Sections were then washed with TBS, counterstained with 4′,6‐diamidino‐2‐phenylindole (DAPI, 1 ug/mL), washed with water, and then mounted with a glycerol based mounting media. Representative fluorescent images were taken at ×20 using Echo Revolution fluorescent microscope.

### 2.13 Histopathology analysis

The liver, lungs, and a portion of skin tissue contralateral to the tumor from each mouse were fixed overnight in 10% formalin, dehydrated with alcohol, embedded in paraffin wax, deparaffinized, and rehydrated. Tissues were stained with hematoxylin and eosin (H&E) (Fisher HealthCare, Cat#245651, 245827) as previously described by our group (Cranford et al., 2019). Representative histology images were taken using a Keyence microscope. Histological analyses were performed blindly by a certified pathologist (I.C).

### 2.14 Statistical analyses

All data were analyzed using commercial software (GraphPad Software, Prism 7). All in vivo outcomes were analyzed using a two‐way ANOVA where appropriate. If a significant difference was observed, Tukey's multiple comparisons test was performed to further define group differences. All gene expression analyses were analyzed using a Student's *t*‐test. Statistical significance was set with an alpha value of *p* ≤ 0.05. Data are presented as mean ± standard error of mean (SEM). In figures, significant main effects (M.E.) are indicated by a horizontal line, significant interactions and *t*‐tests are indicated by *, and trends are indicated by the exact *p* value. All figures, excluding IVIS, were generated using GraphPad Prism.

## 3 RESULTS

### 3.1 Experimental design and animal characteristics

The experimental design is illustrated in Figure 1a–c. Briefly, 10‐week‐old female BALB/c mice were inoculated with either PBS or 4T1‐Luc2‐RFP cells on the right side of the fourth pair mammary gland fat pads at Week 0, and 2 weeks subsequently either exposed to a sterile cutaneous incision (wounded) or no incision (sham) on the contralateral side of the tumor. Emodin or PBS was administrated via oral gavage 2 days prior to wounded and sham BC mice and three times a week thereafter. A total of six groups were used which are presented in Figure 1c. The incision was allowed to heal to model surgery wound healing.

No differences in body weight were detected over the experimental duration (data not shown). IVIS imaging (in vivo) was performed to visualize the tumors 2 weeks following tumor inoculation; there were no differences in tumor growth prior to surgery wounding (Figure 1d). Mice were uniformly categorized based on the bioluminescence of luciferase supported 4T1‐Luc2‐RFP tumor cells. There were no indications of infection at the wound in any mice over the study period. At completion of the study, the wound was evaluated by a pathologist; there were no signs of infection or inflammation in any group and no discernible variation in the healing course (Figure 1e). Additionally, the liver was assessed and emodin treatment, along with all other groups, did not exhibit any signs of liver toxicity (Figure 1e), which is consistent with our previous report regarding the safety of emodin (Sougiannis et al., 2021).

### 3.2 Emodin reduces surgical wounding‐accelerated breast tumor progression

**FIGURE 2:**
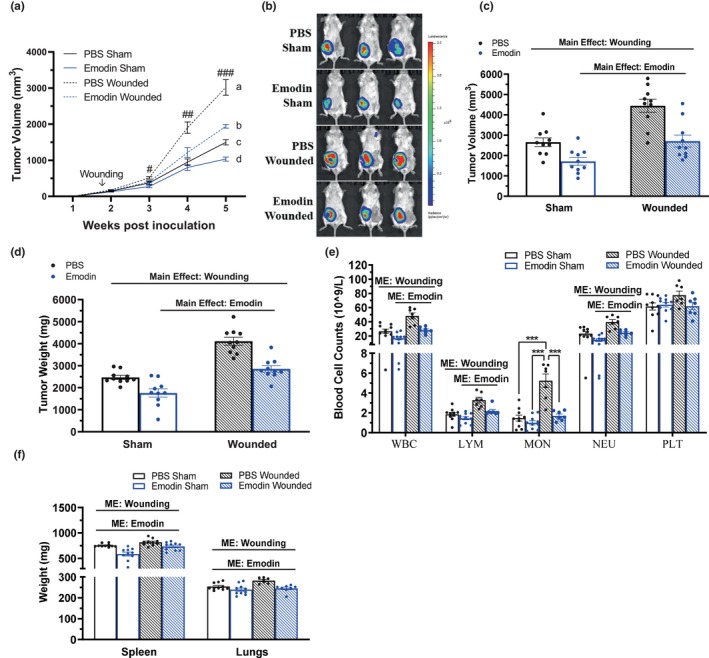
Emodin reduces surgical wounding accelerated breast tumor progression and reduces increased circulating white blood cells. Sham and wounded breast tumor bearing female BALB/c mice were euthanized 5 weeks post 4T1‐Luc2‐RFP cell inoculation. (a) Weekly tumor volume palpations given in millimeters^3^ (mm^3^) (*n* = 10/group). Groups not containing the same letters indicate statistical significance between groups (*p* ≤ 0.05) using a two‐way mixed ANOVA with a Tukey post hoc; # indicates a significant difference between emodin sham and PBS wounded, ## indicates a significant difference between PBS wounded and PBS sham, PBS wounded and emodin wounded, and PBS wounded and emodin sham, ### indicates that all groups are significant from one another. (b) Representative bioluminescent images of luciferase carried 4T1‐Luc2‐RFP tumor cells using in vivo IVIS before euthanasia. Tumors excised from sham and wounded mice for (c) tumor volume given in millimeters^3^ (mm^3^) (*n* = 10/group) and (d) tumor weight in milligrams (mg) (*n* = 10/group). (e) Circulating white blood cells (WBCs), lymphocytes (LYM), monocytes (MON), neutrophils (NEU), and platelets (PLT) determined in whole blood using VetScan HM5 (*n* = 7–10/group). (f) Spleen and lungs excised from sham and wounded mice for weight given in milligrams (mg) (*n* = 7‐10/group). Significance was determined using a two‐way ANOVA (*p* ≤ 0.05). ‘ME’ indicates a main effect between groups. *Indicates a significant interaction (*p* ≤ 0.05) between groups using a Tukey post hoc analysis.

Mice with surgical wounding exhibited significant accelerated tumor growth as indicated by greater tumor volume palpitations at Weeks 4 and 5 following breast cancer cell inoculation (Figure 2a; *p* = 0.0006 at Week 4 and *p* = 0.0002 at Week 5; PBS sham vs. PBS wounded), and this was significantly reduced with emodin treatment at both timepoints (Figure 2a; *p* = 0.0182 at Week 4 and *p* = 0.0034 at Week 5; PBS wounded vs. emodin wounded). Further, and consistent with our previously published work (Iwanowycz, Wang, Hodge, et al., 2016), emodin treatment significantly reduced breast tumor volume in mice without wounding at Week 5 (Figure 2a; *p* = 0.0006; PBS sham vs. emodin sham). These results were confirmed by reduced bioluminescence of luciferase carried 4T1‐Luc2‐RFP breast tumor cells via IVIS in vivo (3 weeks post wounding at ~15 weeks of age; Figure 2b) between emodin‐treated sham and wounded groups compared to that of PBS. Further, at euthanasia, wounding increased both tumor volume (Figure 2c; *p* ≤ 0.0001) and tumor weight (Figure 2d; *p* ≤ 0.0001), which were reduced by emodin (Figure 2c; *p* ≤ 0.0001) (Figure 2d; *p* ≤ 0.0001).

### 3.3 Emodin reduces surgical wounding increased circulating white blood cells

Systemic inflammation has been suggested to mediate surgery‐induced tumor outgrowth (Krall et al., 2018). Wounding increased circulating white blood cells (WBCs; *p* ≤ 0.0001), lymphocytes (LYM; *p* ≤ 0.0001), monocytes (MON; *p* ≤ 0.0001), and neutrophils (NEU; *p* ≤ 0.0001) (Figure 2e) thus promoting systemic inflammation. The use of anti‐inflammatory compound, emodin reduced systemic inflammation as evidenced by reduced elevated circulating WBCs (*p* ≤ 0.0001), LYM (*p* = 0.0008), MON (*p* ≤ 0.0001), and NEU (*p* ≤ 0.0001) (Figure 2e). However, there was an interaction only for MON. Specifically, within PBS (i.e., vehicle) there was an increase in MON with wounding (Figure 2e; *p* ≤ 0.0001; PBS sham vs. PBS wounded) and within wounding there was a decrease in MON with emodin (Figure 2e; *p* ≤ 0.0001; PBS wounded vs. emodin wounded). Further, wounding increased lung (*p* = 0.0125; Figure 2f) and spleen (*p* = 0.0002; Figure 2f) weights, which was reduced by emodin in both the lungs (*p* = 0.0003; Figure 2f) and spleen (*p* ≤ 0.0001; Figure 2f).

### 3.4 Emodin reduces primary breast tumor cell proliferation and enhances apoptosis

**FIGURE 3:**
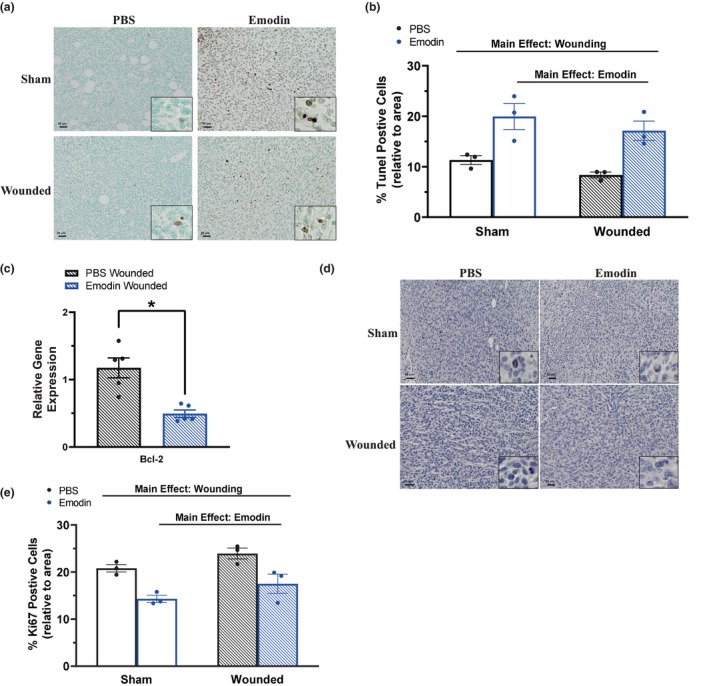
Emodin reduces primary breast tumor cell proliferation and enhances apoptosis. Immunohistochemistry of breast tumor sections from sham and wounded mice *(n* = 3/group) stained with TUNEL for detection of apoptotic cells and Ki67 to examine proliferation. (a) Representative 20x images of breast tumors stained with TUNEL with 40x inserts. Scale bar = 50 μm. (b) Percentage of apoptotic (positively stained TUNEL‐DAB) cells relative to area. (c) Gene expression analysis of Bcl‐2, an anti‐apoptotic marker, within the tumor using qPCR. Data were normalized to PBS wounded and compared with five reference targets (B2M, TBP, HPRT, HMBS, and H2AFV) which were evaluated for expression stability using GeNorm. *Significantly different from control using a Student's *t*‐test (*p* ≤ 0.05). (d) Representative 20x images of breast tumors stained with Ki67 with 40x inserts. Scale bar = 50 μm. (e) Percentage of Ki67‐positive cells (positively stained Ki67‐DAB) cells relative to area. Significance was determined using a two‐way ANOVA (*p* ≤ 0.05).

To examine the antitumoral effects of emodin, breast tumor sections were stained with TUNEL for assessment of apoptotic cells (Figure 3a) and Ki67 to determine proliferation (Figure 3d). The proportion of apoptotic (positively stained) cells were decreased with surgical wounding (Figure 3c; *p* = 0.0404). In support of its antitumor effects, emodin enhanced the proportion of apoptotic (positively stained) cells in the primary breast tumor (Figure 3b; *p* ≤ 0.0001). Further, gene expression of Bcl‐2, an antiapoptotic marker, was significantly decreased in breast tumors of emodin‐wounded mice compared to that of PBS (Figure 3c; *p* = 0.0146). In addition, wounding increased (*p* ≤ 0.0397) and emodin decreased (*p* ≤ 0.001) the proportion of Ki67‐positive cells which is indicative of reduced proliferation of breast cancer cells, likely contributing to emodin's antitumor effects (Figure 3e).

### 3.5 Emodin reduces lung metastasis

**FIGURE 4:**
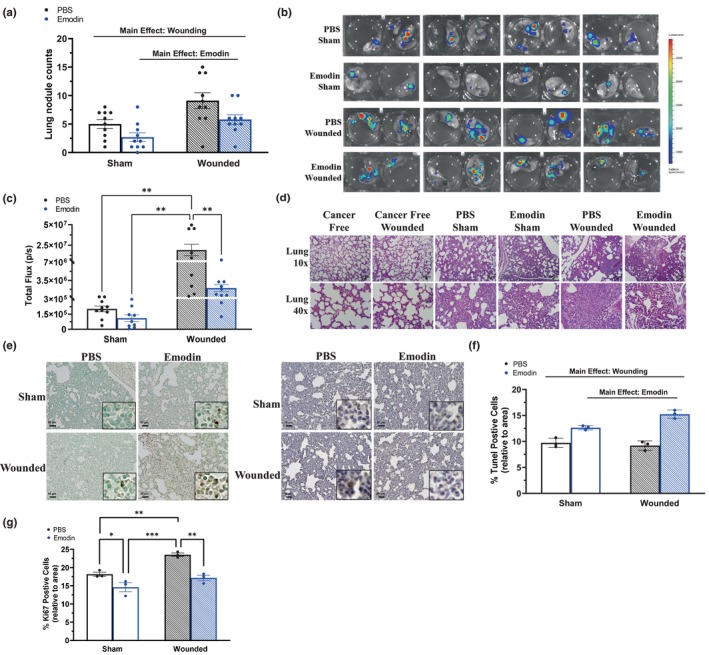
Emodin reduces lung metastasis. Sham and wounded orthotopic breast tumor bearing female BALB/c mice were euthanized 5 weeks post 4T1‐Luc2‐RFP cell inoculation. (a) Lung nodules were determined and counted from IVIS images of ex vivo lungs (*n* = 10/group). (b) Representative bioluminescent images of luciferase carried 4 T1‐Luc2‐RFP tumor cells in ex vivo lungs using IVIS immediately after euthanasia. (c) Quantification of bioluminescence of luciferase carried 4T1‐Luc2‐RFP tumor cells in ex vivo lungs represented by the total flux (radiance (photons/sec) in each pixel summed or integrated over the region of interest area) (*n* = 10/group). Immunohistochemistry of lung sections from sham and wounded mice (*n* = 3/group) stained with TUNEL for detection of apoptotic cells and Ki67 to examine proliferation. (d) Representative 10x and 40x images of lungs stained with H&E. Scale bar = 50 μm. (e) Representative 20x images with 40x inserts of lungs stained with TUNEL (left) and Ki67 (right). Scale bar = 50 μm. (f) Percentage of apoptotic (positively stained TUNEL‐DAB) cells relative to area. (g) Percentage of Ki67‐positive cells (positively stained Ki67‐DAB) cells relative to area. Significance was determined using a two‐way ANOVA (*p* ≤ 0.05). Asterisks indicate a significant interaction between groups using a Tukey post hoc analysis (*indicates *p* ≤ 0.05; **indicates *p* ≤ 0.01; ***indicates *p* ≤ 0.001).

Wounding increased the quantity of nodules on ex vivo lungs (*p* = 0.0065; Figure 4a), which was reduced by emodin (*p* = 0.0007; Figure 4a). To further assess metastasis in the lungs, IVIS was implemented 3 weeks post wounding at ~15 weeks of age to compute the total flux (bioluminescence) of luciferase carried 4T1‐Luc2‐RFP tumor cells (Figure 4b). Consistent with the lung nodule data, wounding increased the total flux of luciferase carried 4T1‐Luc2‐RFP tumor cells (*p* = 0.0036; Figure 4b,c), and this was reduced by emodin (*p* = 0.0142; Figure 4b,c). An interaction revealed that within PBS (i.e., vehicle) there was an increase in lung metastasis signified by increased total flux for IVIS in ex vivo lungs with wounding (Figure 4b,c; *p* = 0.0017) and within wounding there was a decrease in flux with emodin (Figure 4b,c; *p* = 0.0048). Following assessment by a pathologist (I.C.), no specific histological findings within the lungs among cancer free, cancer‐free wounded, emodin sham, and emodin wounded were identified (Figure 4d). On the other hand, both PBS groups exhibited localized histopathological findings including infiltration by neutrophils and other inflammatory cells in PBS sham group. Metastatic foci comprised of tumor cells were only observed in the lungs of PBS wounded mice (Figure 4d).

### 3.6 Emodin enhances apoptosis and reduces proliferation of breast cancer cells within lungs

As in the primary tumor, surgical wounding diminished the proportion of apoptotic (positively stained) cells in the lungs (Figure 4e,f; *p* ≤ 0.0001). In support of emodin's antitumor effects, we report an increase in the percentage of apoptotic (positively stained) cells (Figure 4f; *p* = 0.0488). Consistent with our previous report, wounding increased the proportion of Ki67‐positive cells within lungs compared to sham (Figure 4g; *p* = 0.0012); and as hypothesized, emodin decreased the proportion of Ki67‐positive cells within lungs compared to PBS (Figure 4g; *p* = 0.0003). An interaction revealed that within PBS (i.e., vehicle) there was an increase in the proportion of Ki67‐positive cells with wounding (Figure 4g; *p* = 0.0067) and within wounding there was a decrease in the proportion of Ki67‐positive cells with emodin (Figure 4g; *p* = 0.0023).

### 3.7 Emodin decreases pro‐tumoral M2 macrophages in the primary tumor

**FIGURE 5:**
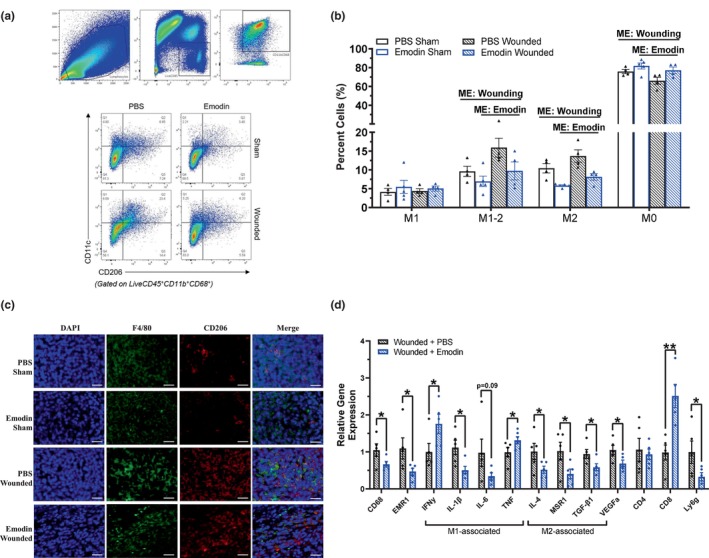
Emodin decreases pro‐tumoral M2 macrophages and downregulates mRNA expression of macrophage markers in the primary tumor. Sham and wounded orthotopic breast tumor bearing female BALB/c mice treated with PBS and emodin were euthanized 5 weeks post 4T1‐Luc2‐RFP cell inoculation. Breast tumors cells were gated for non‐debris singlets and considered live immune cells with ZombieGreen^Neg/Low^ and CD45^+^. From the Live CD45^+^ population, CD11b^+^CD68^+^ cells were identified as macrophages. From the CD11b^+^CD68^+^ macrophage population, CD206^−^CD11c^−^ cells were identified as M0 macrophages; CD206^−^CD11c^+^ as M1 macrophages; CD206^+^CD11c^−^ cells as M2 macrophages; CD206^+^CD11c^+^ as M1‐M2 transitional macrophages. (a) Flow plot identifying live immune cells (CD45^+^), and M0 (CD206^−^CD11c^−^), M1 (CD206^−^CD11c^+^), M2 (CD206^+^CD11c^−^ cells), and M1‐M2 transitional (CD206^+^CD11c^+^) macrophages within breast tumors. (b) Percentages of M1, transitional M1‐M2, M2, and M0 macrophages (*n* = 4/group). Significance was determined using a two‐way ANOVA (*p* ≤ 0.05). ‘ME’ indicates a main effect between groups. (c) Representative 20x images of immunofluorescence staining of F4/80 and CD206 in breast tumor tissues harvested from sham and wounded mice. DAPI (blue) as an individual channel for visualization of nuclei (left), F4/80 (green) as an individual channel, CD206 (red) as an individual channel, and merged (right). Scale bar = 100 μm. (d) qPCR analysis of the following markers: monocyte/macrophage (CD68 and EMR1/F480), pro‐inflammatory M1 macrophage markers (IFNy, IL‐1β, IL‐6, and TNF), anti‐inflammatory M2 macrophage markers (IL‐4, MSR1, and TGF‐β1), angiogenesis (VEGFa), T cells (CD4 and CD8), and neutrophils (Ly6G) (*n* = 5/group). For gene expression data, data were normalized to PBS wounded and compared with five reference targets (B2M, TBP, HPRT, HMBS, and H2AFV), which were evaluated for expression stability using GeNorm. Asterisks indicate a significant difference from control using a Student's *t*‐test (*indicates *p* ≤ 0.05; **indicates *p* ≤ 0.01).

To characterize the antitumorigenic effects of emodin linked to surgical wounding, breast tumors were analyzed to assess macrophage populations via flow cytometry. Flow plots (representative) of live immune cells (CD45^+^), and M0 (CD206^−^CD11c^−^), M1 (CD206^−^CD11c^+^), M2 (CD206^+^CD11c^−^ cells), and M1‐M2 transitional (CD206^+^CD11c^+^) macrophages are illustrated in Figure 5a. We document that wounding enhanced the number of transitional M1‐M2 macrophages (CD206^+^CD11c^+^; *p* = 0.0348) and M2 macrophages (CD206^+^; *p* = 0.0196), populations associated with tumor progression and immune suppression, and inhibited the number of M0 macrophages (CD206^−^CD11c^−^; *p* = 0.0436) (Figure 5b). On the contrary, emodin reduced transitional M1‐M2 macrophages (CD206^+^CD11c^+^; *p* = 0.0396) and pro‐tumoral M2 macrophages (CD206^+^; *p* = 0.0003), and increased M0 macrophages (CD206^−^CD11c^−^; *p* = 0.0208) (Figure 5b).

### 3.8 Emodin downregulates mRNA expression of macrophage and pro‐tumoral M2 macrophage markers within the primary tumor

The reduction in pro‐tumoral M2 macrophages exhibited by emodin in both sham and surgically wounded mice compared to that of PBS was established with immunofluorescence staining for pan macrophage marker F4/80 and M2 cell‐surface marker CD206 in the primary breast tumor (Figure 5c). To document emodin's capacity to regulate macrophages within the tumor microenvironment and diminish pro‐tumoral effects related to surgical wounding, mRNA expression of select macrophage markers were assessed in breast tumors via RT‐PCR. Emodin significantly reduced total macrophages as signified by decreased expression of pan macrophage markers EMR1 (F4/80, *p* = 0.050) and CD68 (*p* = 0.0420) compared to PBS in wounded mice (Figure 5d). Consistent with the findings from flow cytometry, emodin significantly reduced expression of M2‐associated macrophage markers IL‐4 (*p* = 0.050), MSR1 (*p* = 0.0304), and TGF‐β (*p* = 0.0117) compared to PBS in wounded mice (Figure 5d). Further, tumors of emodin‐wounded mice exhibited significant increased expression of antitumoral M1 macrophage expression IFNγ (*p* = 0.0481) and TNF‐α (*p* = 0.0401); however, reduced expression of IL‐1β (*p* = 0.0394) and a nonsignificant reduction in the expression of IL‐6 (*p* = 0.0861) was documented in emodin‐wounded mice compared to PBS wounded mice (Figure 5d). Consistent with our blood data, we report that emodin decreases neutrophils, important drivers of lung metastatic formation in mouse BC models (Wculek & Malanchi, 2015), in the primary tumor indicated by significantly decreased expression of lymphocyte antigen 6 complex locus G6D (Ly6G; *p* = 0.0010) compared to PBS in wounded mice (Figure 5d). In further support for emodin's antitumoral effects, the angiogenesis marker VEGF was significantly decreased (*p* = 0.050) with perioperative emodin in wounded mice compared to that of PBS (Figure 5d). Finally, the cytotoxic T‐cell marker CD8, important in promoting antitumor immune responses, inhibiting metastasis, and correlated with improved prognosis in BC (Joseph et al., 2021; Mahmoud et al., 2011), was significantly increased with perioperative emodin compared to PBS in wounded mice (Figure 5d; *p* = 0.0051).

### 3.9 Emodin downregulates mRNA expression of macrophage and pro‐tumoral M2 macrophage markers in the lungs

**FIGURE 6:**
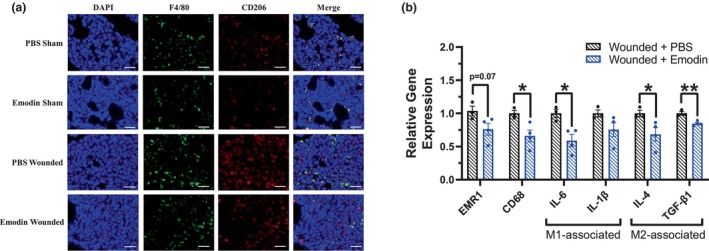
Emodin decreases macrophage number and downregulates mRNA expression of select macrophage markers in lungs. Sham and wounded orthotopic breast tumor bearing female BALB/c mice treated with either PBS or emodin were euthanized 5 weeks post 4T1‐Luc2‐RFP cell inoculation. (a) Representative 20x images of immunofluorescence staining of F4/80 and CD206 in lung tissues harvested from sham and wounded mice treated with PBS or emodin. DAPI (blue) as an individual channel for visualization of nuclei (left), F4/80 (green) as an individual channel, CD206 (red) as an individual channel, and merged (right). Scale bar = 100 μm. (b) qPCR analysis of the following markers: monocyte/macrophage (EMR1/F480), pro‐inflammatory M1 macrophage markers (IL‐1β and IL‐6), and anti‐inflammatory M2 macrophage markers (IL‐4 and TGF‐β1) (*n* = 3‐4/group). For gene expression data, data were normalized to PBS wounded and compared with five reference targets (B2M, TBP, HPRT, HMBS, and H2AFV), which were evaluated for expression stability using GeNorm. *Significantly different from control using a Student's *t*‐test (*p* ≤ 0.05).

Immunofluorescence staining for pan macrophage marker F4/80 and M2 cell‐surface marker CD206 in the lungs revealed reduced macrophages and more specifically reduced M2 associated macrophages in emodin‐treated mice compared to their PBS counterparts in both sham and wounded mice (Figure 6a). Further, emodin reduced macrophage expression of pan macrophage markers EMR1 (F4/80, *p* = 0.0719) and CD68 (*p* = 0.0287) compared to PBS in wounded mice (Figure 6b). Emodin also significantly reduced expression of M2‐associated macrophage markers IL‐4 (*p* = 0.050) and TGF‐β compared to PBS in wounded mice (*p* = 0.0042) (Figure 6b). As in the primary tumor, we also report that select M1 macrophage markers were reduced with emodin in wounded mice; emodin‐wounded mice exhibited reduced expression of IL‐6 (*p* = 0.0188) and a reduction in IL‐1β (*p* = 0.1193) compared to that of PBS (Figure 6b).

## 4 DISCUSSION

For patients with TNBC, metastasis to the lungs or bones is the leading cause of mortality after preliminary success of surgery and/or anticancer therapeutics (Waks & Winer, 2019). It is becoming more evident that tumor resection surgery and the succeeding wound healing response contribute to early metastatic relapse (Dillekas et al., 2014, 2016; Tohme et al., 2017). Indeed, we recently reported that the surgical wounding procedure itself, at a remote site and in the absence of tumor resection, is adequate to hasten primary tumor progression and promote lung metastasis in a murine model of TNBC (McDonald, VanderVeen, Bullard, et al., 2022). This is of great concern given that surgical resection is a mainstay of BC therapy. Thus, in the current study we sought to examine emodin's potential as a perioperative treatment to mitigate surgical wounding augmented tumor growth and lung metastasis. We demonstrate that emodin indeed inhibits postsurgical wounding tumor outgrowth and metastasis to lungs in a TNBC model and this was associated with increased apoptosis and decreased proliferation in both the primary tumor and in the lungs. We further demonstrate that emodin's antitumor responses are consistent with its inhibitory effect on macrophages, especially pro‐tumor M2 macrophages.

Clinical studies have demonstrated that interventions given during the fragile perioperative period greatly influence cancer recurrence and survival (Forget et al., 2010; Retsky et al., 2013). For instance, perioperative administration of nonsteroidal anti‐inflammatory drugs (NSAIDs) reduces the incidence of early metastatic recurrence in BC patients (Forget et al., 2010; Retsky et al., 2013) and the NSAID meloxicam prevents surgery‐induced tumor outgrowth in murine BC models (Krall et al., 2018). However, NSAIDs may cause immunosuppression, wound healing delay, and other severe side effects (Shaji et al., 2021); thus, safer anti‐inflammatory interventions are needed for this clinical application. Our group has previously reported that emodin can prevent breast tumor growth and lung metastasis in mice (Iwanowycz, Wang, Hodge, et al., 2016; Jia et al., 2014; Liu et al., 2020). In the current study, we advance these findings to demonstrate that emodin can mitigate surgical wounding‐accelerated tumor growth and lung metastasis; not only did emodin reduce growth of the primary tumor but it also reduced metastasis associated with wound healing. Emodin has been reported to exert its antitumor effects, at least in part, through inducing apoptosis and suppressing proliferation as recent evidence suggests (McDonald, VanderVeen, Velazquez, et al., 2022). Our findings support this notion, as we demonstrate that emodin promotes apoptosis and inhibits proliferation of cancer cells in the primary tumor and in the lungs, likely contributing, at least in part, to the observed reduction in BC growth and metastasis in the lungs. However, additional work is needed to determine the contribution of emodin's actions on apoptosis and proliferation to the documented reduction in BC growth and metastasis.

Alterations in the number and function of peripheral blood cells can correlate with clinical outcome (Wang et al., 2020) and thus play an essential role in the diagnosis and treatment of BC (Chen et al., 2020). For instance, increased WBCs and neutrophils have consistently been associated with enhanced tumor growth and lung metastasis, shorter disease‐free and overall survival time, and poor prognosis in both preclinical and clinical studies (Chen et al., 2020; Orditura et al., 2016; Wang et al., 2015). Interestingly, an increase in WBCs is the proposed mechanism whereby surgical wounding can actually increase metastasis in the lungs. Indeed, it has been reported that surgery‐induced tumor outgrowth is associated with the deployment of myeloid cells into the circulation of wounded mice (Krall et al., 2018); in particular, inflammatory monocytes, which differentiate into macrophages in the tumor microenvironment, have been implicated as the likely key mediator of the systemic response to surgery (Krall et al., 2018). Our previous data support this notion given that surgical wounding was associated with increased WBCs and specifically neutrophils and monocytes (McDonald, VanderVeen, Bullard, et al., 2022). Excitingly, in the current study, emodin reduced the surgery‐induced elevation in WBCs, lymphocytes, monocytes, and neutrophils in wounded mice. However, whether this decrease in WBCs with emodin is reflective of emodin's direct action on the wound itself, or a result of its actions on tumor growth, or both, could not be concluded from the current study. Future studies that examine emodin's actions on the peripheral immune response over time following wounding are necessary to establish the precise mechanism.

It has previously been documented that monocytes, predecessors of macrophages, are summoned into circulation succeeding surgical wounding, intensifying their accessibility for enrollment into tumors and signifying a means whereby surgical wounding may enhance metastasis to the lungs (Krall et al., 2018). Our previous work lends credence to this as we demonstrated that surgical wounding does indeed enhance macrophages in the primary tumor and in the lungs, and specifically M2‐like pro‐tumor macrophages or tumor‐associated macrophages (TAMs) (McDonald, VanderVeen, Bullard, et al., 2022). TAMs have been widely reported to promote tumor development and metastasis and in multiple cancer models through driving tumor cell proliferation and invasiveness, enhancing angiogenesis, promoting immunosuppression, and enabling cancer stem cell formation and maintenance (Medrek et al., 2012; Qian & Pollard, 2010; Rudnick & Kuperwasser, 2012). Given their important role in all stages of cancer promotion, TAMs have been deliberated as a target for BC. However, macrophages have not yet been utilized as a target for deterrence of BC lung metastasis. Our labs have discovered that emodin has context‐dependent bidirectional effects on macrophage activation and can inhibit BC growth and lung metastasis by reducing recruitment and M2‐like polarization of TAMs (Iwanowycz, Wang, Hodge, et al., 2016; Jia et al., 2014). The current study extends these findings to demonstrate that emodin can prevent the increase in pro‐tumoral M2 macrophages as well as transitional (M1‐M2) macrophages in both the primary tumor and in the lungs of mice that were wounded. We have recently documented that emodin can inhibit expression of P2X7 (Sougiannis et al., 2022)—a receptor on macrophages that has been reported to promote inflammation and that can be targeted by emodin (Jelassi et al., 2013; Sougiannis et al., 2022). Thus, it is possible that emodin may be antagonizing the P2X7 receptor, which may be a mechanism whereby emodin is mediating its actions on macrophages in the current study. Overall, these findings suggest that emodin may alleviate the surgery induced accelerated metastasis of BC to the lungs by its inhibitory actions on macrophages, more specifically M2 macrophages.

The effects of emodin on macrophages were further established by gene expression in both the primary breast tumors and the lungs. Emodin‐treated wounded mice had downregulated mRNA gene expression of pan‐macrophage markers EMRI (F480) and CD68, and M2 macrophage markers Arg1, IL‐4, IL‐10, IL‐13, MSR1, and TGFβ1. M2‐associated markers are critical regulators of the tumor microenvironment and have been incriminated in governing all attributes of tumor progression including survival, proliferation, metastasis, and cancer cell evasion (Kwaśniak et al., 2019; Mirlekar, 2022). A recent review and supporting literature boast that targeting these M2 markers can reduce primary breast tumor burden and prevent BC lung metastasis (Munir et al., 2021; Papageorgis et al., 2015; Venmar et al., 2014). Further, in support of our findings that emodin increases expression of select M1 macrophage markers (IFNγ and TNF) in wounded mice, M1 macrophages and their associated cytokines in the tumor‐associated environment (TME) have been consistently associated with reduced breast tumor burden and aggressiveness given their tumoricidal functions (Munir et al., 2021). Further, we document a significant reduction in VEGF gene expression in emodin‐wounded mice indicating a potential decrease in angiogenesis. Finally, the expression of neutrophil marker Ly6g, an important driver of lung metastatic colonization (Wculek & Malanchi, 2015), was inhibited in the tumor and lungs of emodin‐treated wounded mice, and CD8, a marker of cytotoxic T cells was increased in the primary tumor with emodin. While the primary focus of the study was on macrophages, emodin's actions on neutrophils and cytotoxic T cells should be further explored. Together with the cellular data, the gene expression data suggest that emodin may create a more favorable immune environment to inhibit surgery‐induced accelerated primary tumor growth and lung metastasis.

We recognize that there are several limitations to the current study. We used only one mouse model of BC, specifically TNBC, which does not represent of all subtypes of BC. Further, while our data indicate that emodin's ability to reduce surgical wounding‐accelerated BC lung metastasis may be mediated via its actions on macrophages, particularly M2 macrophages, studies using macrophage manipulation techniques are needed to convincingly establish macrophages as meditators of emodin's impact. Our immune findings and consequent interpretations are restricted to one study time‐point (i.e., endpoint); evaluation of preceding time‐points would likely add to our understanding of emodin's actions on the stimulus (i.e., wounding or tumor growth) and subsequent mediation of the increase in inflammatory monocytes, the infiltration of TAMs, and the promotion of lung metastasis. The 4T1‐luciferase2‐red fluorescent protein (modified‐4T1), intentionally utilized in this study given that it portrays diminished lung metastatic incidence in contrast with the prompt growth and metastasis of parental 4T1 breast tumors (Atiya et al., 2019) may promote an immune response against luciferase, subsequently confining tumor invasiveness. This has not been evaluated in our laboratory; however, others have documented such response (Baklaushev et al., 2017), which imposes constraints to this model. Finally, we acknowledge that the study design did not allow us to determine whether the effects of emodin on lung metastasis are reflective of emodin's actions on reducing the primary tumor, a direct effect of emodin on lung metastasis, or both; future studies are needed to parse this out.

In conclusion, the current data support the natural compound emodin as an effective therapy to be used perioperatively to alleviate the surgery triggered promotion of tumor outgrowth and lung metastasis in TNBC, likely through its inhibitory actions on inflammatory monocytes and macrophages. This is of relevance given that there are no preventive agents for ER‐BC, including TNBC which characteristically has higher metastatic recurrence rates and shorter overall and disease‐free survival (Waks & Winer, 2019). However, additional preclinical work as well as clinical trials will be required for successful development of emodin in this regard to include appropriate timing and dosing of emodin to achieve best efficacy.

## AUTHOR CONTRIBUTIONS

Conceptualization, S.J.M., B.N.V., D.F., E.A.M.; Formal Analysis, S.J.M., B.M.B., B.N.V., T.D.C., I.C.; Funding acquisition, S.J.M., D.F., E.A.M.; Investigation, S.J.M., B.M.B., B.N.V., T.D.C., I.C., D.F., E.A.M.; Methodology, S.J.M., B.M.B., B.N.V., T.D.C., I.C., D.F., E.A.M.; Supervision, D.F., E.A.M.; Writing – original draft, S.J.M.; Writing – review and editing, S.J.M., B.M.B., B.N.V., T.D.C., I.C., D.F., E.A.M.; All authors have read and agreed to the published version of the manuscript.

## ETHICS STATEMENT

The Institutional Animal Care and Usage Committee of the University of South Carolina approved all experiments, and all methods were performed in accordance with the American Association for Laboratory Animal Science.

## FUNDING INFORMATION

This research was supported by grants from the National Institutes of Health: R01CA218578 (to D.F. and E.A.M.), 3R01CA218578‐03S1 (to S.J.M.), U01CA272977 (to E.A.M.), and UT1AA 030690 (to D.F.).

## CONFLICT OF INTEREST STATEMENT

This work was supported in part by a small business innovation research grant to AcePre, LLC. B.N.V., D.F., E.A.M. are affiliated with AcePre, LLC. The funding agency took no part in the collection, analysis, interpretation of the data, or the writing of the manuscript.

## Data Availability

The authors confirm that the data supporting the findings of this study are available within the article. Further information about the data is available upon reasonable request.

## References

[phy215813-bib-0001] Atiya, H. I. , Dvorkin‐Gheva, A. , Hassell, J. , Patel, S. , Parker, R. L. , Hartstone‐Rose, A. , Hodge, J. , Fan, D. , & Ramsdell, A. F. (2019). Intraductal adaptation of the 4T1 mouse model of breast cancer reveals effects of the epithelial microenvironment on tumor progression and metastasis. Anticancer Research, 39(5), 2277–2287. 31092419 10.21873/anticanres.13344 PMC7339129

[phy215813-bib-0002] Baklaushev, V. P. , Kilpeläinen, A. , Petkov, S. , Abakumov, M. A. , Grinenko, N. F. , Yusubalieva, G. M. , Latanova, A. A. , Gubskiy, I. L. , Zabozlaev, F. G. , Starodubova, E. S. , Abakumova, T. O. , Isaguliants, M. G. , & Chekhonin, V. P. (2017). Luciferase expression allows bioluminescence imaging but imposes limitations on the Orthotopic mouse (4T1) model of breast cancer. Scientific Reports, 7(1), 7715. 28798322 10.1038/s41598-017-07851-z PMC5552689

[phy215813-bib-0003] Baum, M. , Chaplain, M. A. J. , Anderson, A. R. A. , Douek, M. , & Vaidya, J. S. (1999). Does breast cancer exist in a state of chaos? European Journal of Cancer, 35(6), 886–891. 10533467 10.1016/s0959-8049(99)00067-2

[phy215813-bib-0004] Chen, L. , Kong, X. , Yan, C. , Fang, Y. , & Wang, J. (2020). The research progress on the prognostic value of the common hematological parameters in peripheral venous blood in breast cancer. Oncotargets and Therapy, 13, 1397–1412. 32104003 10.2147/OTT.S227171 PMC7028387

[phy215813-bib-0005] Cheng, L. , Swartz, M. D. , Zhao, H. , Kapadia, A. S. , Lai, D. , Rowan, P. J. , Buchholz, T. A. , & Giordano, S. H. (2012). Hazard of recurrence among women after primary breast cancer treatment—a 10‐year follow‐up using data from SEER‐Medicare. Cancer Epidemiology, Biomarkers & Prevention, 21(5), 800–809. 10.1158/1055-9965.EPI-11-1089 22426147

[phy215813-bib-0006] Cole, B. F. , Gelber, R. D. , Gelber, S. , Coates, A. S. , & Goldhirsch, A. (2001). Polychemotherapy for early breast cancer: An overview of the randomised clinical trials with quality‐adjusted survival analysis. Lancet, 358(9278), 277–286. 11498214 10.1016/S0140-6736(01)05483-6

[phy215813-bib-0007] Colleoni, M. , Sun, Z. , Price, K. N. , Karlsson, P. , Forbes, J. F. , Thürlimann, B. , Gianni, L. , Castiglione, M. , Gelber, R. D. , Coates, A. S. , & Goldhirsch, A. (2016). Annual Hazard rates of recurrence for breast cancer during 24 years of follow‐up: Results from the international breast cancer study group trials I to V. Journal of Clinical Oncology, 34(9), 927–935. 26786933 10.1200/JCO.2015.62.3504 PMC4933127

[phy215813-bib-0008] Cranford, T. L. , Velázquez, K. T. , Enos, R. T. , Sougiannis, A. T. , Bader, J. E. , Carson, M. S. , Bellone, R. R. , Chatzistamou, I. , Nagarkatti, M. , & Murphy, E. A. (2019). Effects of high fat diet‐induced obesity on mammary tumorigenesis in the PyMT/MMTV murine model. Cancer Biology & Therapy, 20(4), 487–496. 30388923 10.1080/15384047.2018.1537574 PMC6422456

[phy215813-bib-0009] Demicheli, R. , Retsky, M. W. , Hrushesky, W. J. M. , & Baum, M. (2007). Tumor dormancy and surgery‐driven interruption of dormancy in breast cancer: Learning from failures. Nature Clinical Practice. Oncology, 4(12), 699–710. 10.1038/ncponc0999 18037874

[phy215813-bib-0010] Dillekas, H. , Demicheli, R. , Ardoino, I. , Jensen, S. A. , Biganzoli, E. , & Straume, O. (2016). The recurrence pattern following delayed breast reconstruction after mastectomy for breast cancer suggests a systemic effect of surgery on occult dormant micrometastases. Breast Cancer Research and Treatment, 158(1), 169–178. 27306422 10.1007/s10549-016-3857-1 PMC4937089

[phy215813-bib-0011] Dillekas, H. , Transeth, M. , Pilskog, M. , Assmus, J. , & Straume, O. (2014). Differences in metastatic patterns in relation to time between primary surgery and first relapse from breast cancer suggest synchronized growth of dormant micrometastases. Breast Cancer Research and Treatment, 146(3), 627–636. 25038878 10.1007/s10549-014-3057-9 PMC4112046

[phy215813-bib-0012] Enos, R. T. , Davis, J. M. , Velázquez, K. T. , McClellan, J. L. , Day, S. D. , Carnevale, K. A. , & Murphy, E. A. (2013). Influence of dietary saturated fat content on adiposity, macrophage behavior, inflammation, and metabolism: Composition matters. Journal of Lipid Research, 54(1), 152–163. 23103474 10.1194/jlr.M030700 PMC3520521

[phy215813-bib-0013] Enos, R. T. , Velázquez, K. T. , McClellan, J. L. , Cranford, T. L. , Walla, M. D. , & Murphy, E. A. (2015). Lowering the dietary omega‐6: Omega‐3 does not hinder nonalcoholic fatty‐liver disease development in a murine model. Nutrition Research, 35(5), 449–459. 25934114 10.1016/j.nutres.2015.04.003 PMC4451001

[phy215813-bib-0014] Fisher, B. , & Fisher, E. R. (1959). Experimental studies of factors influencing hepatic metastases. III. Effect of surgical trauma with special reference to liver injury. Annals of Surgery, 150, 731–744. 13823186 10.1097/00000658-195910000-00015 PMC1613460

[phy215813-bib-0015] Forget, P. , Vandenhende, J. , Berliere, M. , Machiels, J. P. , Nussbaum, B. , Legrand, C. , & de Kock, M. (2010). Do intraoperative analgesics influence breast cancer recurrence after mastectomy? A retrospective analysis. Anesthesia and Analgesia, 110(6), 1630–1635. 20435950 10.1213/ANE.0b013e3181d2ad07

[phy215813-bib-0016] Gianni, L. , Dafni, U. , Gelber, R. D. , Azambuja, E. , Muehlbauer, S. , Goldhirsch, A. , Untch, M. , Smith, I. , Baselga, J. , Jackisch, C. , Cameron, D. , Mano, M. , Pedrini, J. L. , Veronesi, A. , Mendiola, C. , Pluzanska, A. , Semiglazov, V. , Vrdoljak, E. , Eckart, M. J. , … Herceptin Adjuvant (HERA) Trial Study Team . (2011). Treatment with trastuzumab for 1 year after adjuvant chemotherapy in patients with HER2‐positive early breast cancer: A 4‐year follow‐up of a randomised controlled trial. The Lancet Oncology, 12(3), 236–244. 21354370 10.1016/S1470-2045(11)70033-X

[phy215813-bib-0017] Grotto, D. , & Zied, E. (2010). The standard American diet and its relationship to the health status of Americans. Nutrition in Clinical Practice, 25(6), 603–612. 21139124 10.1177/0884533610386234

[phy215813-bib-0018] Iwanowycz, S. , Wang, J. , Altomare, D. , Hui, Y. , & Fan, D. (2016). Emodin bidirectionally modulates macrophage polarization and epigenetically regulates macrophage memory. The Journal of Biological Chemistry, 291(22), 11491–11503. 27008857 10.1074/jbc.M115.702092 PMC4882421

[phy215813-bib-0019] Iwanowycz, S. , Wang, J. , Hodge, J. , Wang, Y. , Yu, F. , & Fan, D. (2016). Emodin inhibits breast cancer growth by blocking the tumor‐promoting feedforward loop between cancer cells and macrophages. Molecular Cancer Therapeutics, 15(8), 1931–1942. 27196773 10.1158/1535-7163.MCT-15-0987 PMC4975665

[phy215813-bib-0020] Jelassi, B. , Anchelin, M. , Chamouton, J. , Cayuela, M. L. , Clarysse, L. , Li, J. , Goré, J. , Jiang, L. H. , & Roger, S. (2013). Anthraquinone emodin inhibits human cancer cell invasiveness by antagonizing P2X7 receptors. Carcinogenesis, 34(7), 1487–1496. 23524196 10.1093/carcin/bgt099

[phy215813-bib-0021] Jia, X. , Yu, F. , Wang, J. , Iwanowycz, S. , Saaoud, F. , Wang, Y. , Hu, J. , Wang, Q. , & Fan, D. (2014). Emodin suppresses pulmonary metastasis of breast cancer accompanied with decreased macrophage recruitment and M2 polarization in the lungs. Breast Cancer Research and Treatment, 148(2), 291–302. 25311112 10.1007/s10549-014-3164-7 PMC4224983

[phy215813-bib-0022] Jin, L. , Han, B. , Siegel, E. , Cui, Y. , Giuliano, A. , & Cui, X. (2018). Breast cancer lung metastasis: Molecular biology and therapeutic implications. Cancer Biology & Therapy, 19(10), 858–868. 29580128 10.1080/15384047.2018.1456599 PMC6300341

[phy215813-bib-0023] Joseph, R. , Soundararajan, R. , Vasaikar, S. , Yang, F. , Allton, K. L. , Tian, L. , den Hollander, P. , Isgandarova, S. , Haemmerle, M. , Mino, B. , Zhou, T. , Shin, C. , Martinez‐Paniagua, M. , Sahin, A. A. , Rodriguez‐Canales, J. , Gelovani, J. , Chang, J. T. , Acharya, G. , Sood, A. K. , … Mani, S. A. (2021). CD8+ T cells inhibit metastasis and CXCL4 regulates its function. British Journal of Cancer, 125(2), 176–189. 33795809 10.1038/s41416-021-01338-5 PMC8292398

[phy215813-bib-0024] Klein, C. A. (2020). Cancer progression and the invisible phase of metastatic colonization. Nature Reviews. Cancer, 20(11), 681–694. 33024261 10.1038/s41568-020-00300-6

[phy215813-bib-0025] Krall, J. A. , Reinhardt, F. , Mercury, O. A. , Pattabiraman, D. R. , Brooks, M. W. , Dougan, M. , Lambert, A. W. , Bierie, B. , Ploegh, H. L. , Dougan, S. K. , & Weinberg, R. A. (2018). The systemic response to surgery triggers the outgrowth of distant immune‐controlled tumors in mouse models of dormancy. Science Translational Medicine, 10(436), eaan3464. 29643230 10.1126/scitranslmed.aan3464 PMC6364295

[phy215813-bib-0026] Kwaśniak, K. , Czarnik‐Kwaśniak, J. , Maziarz, A. , Aebisher, D. , Zielińska, K. , Karczmarek‐Borowska, B. , & Tabarkiewicz, J. (2019). Scientific reports concerning the impact of interleukin 4, interleukin 10 and transforming growth factor beta on cancer cells. Central European Journal of Immunology, 44(2), 190–200. 31530989 10.5114/ceji.2018.76273 PMC6745546

[phy215813-bib-0027] Liu, Q. , Hodge, J. , Wang, J. , Wang, Y. , Wang, L. , Singh, U. P. , Li, Y. , Yao, Y. , Wang, D. , Ai, W. , Nagarkatti, P. , Chen, H. , Xu, P. , Murphy, E. A. , & Fan, D. (2020). Emodin reduces breast cancer lung metastasis by suppressing macrophage‐induced breast cancer cell epithelial‐mesenchymal transition and cancer stem cell formation. Theranostics, 10(18), 8365–8381. 32724475 10.7150/thno.45395 PMC7381725

[phy215813-bib-0028] Mahmoud, S. M. A. , Paish, E. C. , Powe, D. G. , Macmillan, R. D. , Grainge, M. J. , Lee, A. H. S. , Ellis, I. O. , & Green, A. R. (2011). Tumor‐infiltrating CD8<sup>+</sup> lymphocytes predict clinical outcome in breast cancer. Journal of Clinical Oncology, 29(15), 1949–1955. 21483002 10.1200/JCO.2010.30.5037

[phy215813-bib-0029] McDonald, S. J. , VanderVeen, B. N. , Bullard, B. M. , Cardaci, T. D. , Madero, S. S. , Chatzistamou, I. , Fan, D. , & Murphy, E. A. (2022). Surgical wounding enhances pro‐tumor macrophage responses and accelerates tumor growth and lung metastasis in a triple negative breast cancer mouse model. Physiological Reports, 10(21), e15497. 36325601 10.14814/phy2.15497 PMC9630756

[phy215813-bib-0030] McDonald, S. J. , VanderVeen, B. N. , Velazquez, K. T. , Enos, R. T. , Fairman, C. M. , Cardaci, T. D. , Fan, D. , & Murphy, E. A. (2022). Therapeutic potential of emodin for gastrointestinal cancers. Integrative Cancer Therapies, 21, 15347354211067469. 34984952 10.1177/15347354211067469 PMC8738880

[phy215813-bib-0031] Medrek, C. , Pontén, F. , Jirström, K. , & Leandersson, K. (2012). The presence of tumor associated macrophages in tumor stroma as a prognostic marker for breast cancer patients. BMC Cancer, 12, 306. 22824040 10.1186/1471-2407-12-306 PMC3414782

[phy215813-bib-0032] Mirlekar, B. (2022). Tumor promoting roles of IL‐10, TGF‐beta, IL‐4, and IL‐35: Its implications in cancer immunotherapy. SAGE Open Medicine, 10, 20503121211069012. 35096390 10.1177/20503121211069012 PMC8793114

[phy215813-bib-0033] Munir, M. T. , Kay, M. K. , Kang, M. H. , Rahman, M. M. , al‐Harrasi, A. , Choudhury, M. , Moustaid‐Moussa, N. , Hussain, F. , & Rahman, S. M. (2021). Tumor‐associated macrophages as multifaceted regulators of breast tumor growth. International Journal of Molecular Sciences, 22(12), 6526. 34207035 10.3390/ijms22126526 PMC8233875

[phy215813-bib-0034] Orditura, M. , Galizia, G. , Diana, A. , Saccone, C. , Cobellis, L. , Ventriglia, J. , Iovino, F. , Romano, C. , Morgillo, F. , Mosca, L. , Diadema, M. R. , Lieto, E. , Procaccini, E. , de Vita, F. , & Ciardiello, F. (2016). Neutrophil to lymphocyte ratio (NLR) for prediction of distant metastasis‐free survival (DMFS) in early breast cancer: A propensity score‐matched analysis. ESMO Open, 1(2), e000038. 27843594 10.1136/esmoopen-2016-000038 PMC5070254

[phy215813-bib-0035] Papageorgis, P. , Ozturk, S. , Lambert, A. W. , Neophytou, C. M. , Tzatsos, A. , Wong, C. K. , Thiagalingam, S. , & Constantinou, A. I. (2015). Targeting IL13Ralpha2 activates STAT6‐TP63 pathway to suppress breast cancer lung metastasis. Breast Cancer Research, 17, 98. 26208975 10.1186/s13058-015-0607-y PMC4531803

[phy215813-bib-0036] Patel, H. K. , & Bihani, T. (2018). Selective estrogen receptor modulators (SERMs) and selective estrogen receptor degraders (SERDs) in cancer treatment. Pharmacology & Therapeutics, 186, 1–24. 29289555 10.1016/j.pharmthera.2017.12.012

[phy215813-bib-0037] Qian, B. Z. , & Pollard, J. W. (2010). Macrophage diversity enhances tumor progression and metastasis. Cell, 141(1), 39–51. 20371344 10.1016/j.cell.2010.03.014 PMC4994190

[phy215813-bib-0038] Retsky, M. , Demicheli, R. , Hrushesky, W. , Forget, P. , Kock, M. , Gukas, I. , Rogers, R. , Baum, M. , Sukhatme, V. , & Vaidya, J. (2013). Reduction of breast cancer relapses with perioperative non‐steroidal anti‐inflammatory drugs: New findings and a review. Current Medicinal Chemistry, 20(33), 4163–4176. 23992307 10.2174/09298673113209990250 PMC3831877

[phy215813-bib-0039] Retsky, M. W. , Demicheli, R. , Hrushesky, W. J. , Baum, M. , & Gukas, I. D. (2008). Dormancy and surgery‐driven escape from dormancy help explain some clinical features of breast cancer. APMIS: Acta Pathologica, Microbiologica, et Immunologica Scandinavica, 116(7–8), 730–741. 18834415 10.1111/j.1600-0463.2008.00990.x

[phy215813-bib-0040] Rizzardi, A. E. , Johnson, A. T. , Vogel, R. I. , Pambuccian, S. E. , Henriksen, J. , Skubitz, A. P. N. , Metzger, G. J. , & Schmechel, S. C. (2012). Quantitative comparison of immunohistochemical staining measured by digital image analysis versus pathologist visual scoring. Diagnostic Pathology, 7, 42. 22515559 10.1186/1746-1596-7-42 PMC3379953

[phy215813-bib-0041] Rudnick, J. A. , & Kuperwasser, C. (2012). Stromal biomarkers in breast cancer development and progression. Clinical & Experimental Metastasis, 29(7), 663–672. 22684404 10.1007/s10585-012-9499-8

[phy215813-bib-0042] Shaji, S. , Smith, C. , & Forget, P. (2021). Perioperative NSAIDs and long‐term outcomes after cancer surgery: A systematic review and meta‐analysis. Current Oncology Reports, 23(12), 146. 34748112 10.1007/s11912-021-01133-8 PMC8575753

[phy215813-bib-0043] Siegel, R. L. , Miller, K. D. , Fuchs, H. E. , & Jemal, A. (2022). Cancer statistics, 2022. CA: A Cancer Journal for Clinicians, 72(1), 7–33. 35020204 10.3322/caac.21708

[phy215813-bib-0044] Sougiannis, A. T. , Enos, R. T. , VanderVeen, B. N. , Velazquez, K. T. , Kelly, B. , McDonald, S. , Cotham, W. , Chatzistamou, I. , Nagarkatti, M. , Fan, D. , & Murphy, E. A. (2021). Safety of natural anthraquinone emodin: An assessment in mice. BMC Pharmacology and Toxicology, 22(1), 9. 33509280 10.1186/s40360-021-00474-1 PMC7845031

[phy215813-bib-0045] Sougiannis, A. T. , VanderVeen, B. , Chatzistamou, I. , Kubinak, J. L. , Nagarkatti, M. , Fan, D. , & Murphy, E. A. (2022). Emodin reduces tumor burden by diminishing M2‐like macrophages in colorectal cancer. American Journal of Physiology. Gastrointestinal and Liver Physiology, 322(3), G383–G395. 35018819 10.1152/ajpgi.00303.2021 PMC8897011

[phy215813-bib-0046] Steiner, J. , Davis, J. M. , McClellan, J. , Enos, R. T. , Carson, J. A. , Fayad, R. , Nagarkatti, M. , Nagarkatti, P. S. , Altomare, D. , Creek, K. E. , & Murphy, E. A. (2014). Dose‐dependent benefits of quercetin on tumorigenesis in the C3(1)/SV40Tag transgenic mouse model of breast cancer. Cancer Biology & Therapy, 15(11), 1456–1467. 25482952 10.4161/15384047.2014.955444 PMC4623114

[phy215813-bib-0047] Tohme, S. , Simmons, R. L. , & Tsung, A. (2017). Surgery for cancer: A trigger for metastases. Cancer Research, 77(7), 1548–1552. 28330928 10.1158/0008-5472.CAN-16-1536 PMC5380551

[phy215813-bib-0048] Venmar, K. T. , Carter, K. J. , Hwang, D. G. , Dozier, E. A. , & Fingleton, B. (2014). IL4 receptor ILR4alpha regulates metastatic colonization by mammary tumors through multiple signaling pathways. Cancer Research, 74(16), 4329–4340. 24947041 10.1158/0008-5472.CAN-14-0093 PMC4134711

[phy215813-bib-0049] Waks, A. G. , & Winer, E. P. (2019). Breast cancer treatment: A review. JAMA, 321(3), 288–300. 30667505 10.1001/jama.2018.19323

[phy215813-bib-0050] Wang, C. , Chen, Y.‐G. , Gao, J.‐L. , Lyu, G.‐Y. , Su, J. , Zhang, Q. , Ji, X. , Yan, J.‐Z. , Qiu, Q.‐L. , Zhang, Y.‐L. , Li, L.‐Z. , Xu, H.‐T. , & Chen, S.‐H. (2015). Low local blood perfusion, high white blood cell and high platelet count are associated with primary tumor growth and lung metastasis in a 4T1 mouse breast cancer metastasis model. Oncology Letters, 10(2), 754–760. 26622565 10.3892/ol.2015.3304 PMC4509112

[phy215813-bib-0051] Wang, L. , Simons, D. L. , Lu, X. , Tu, T. Y. , Avalos, C. , Chang, A. Y. , Dirbas, F. M. , Yim, J. H. , Waisman, J. , & Lee, P. P. (2020). Breast cancer induces systemic immune changes on cytokine signaling in peripheral blood monocytes and lymphocytes. eBioMedicine, 52, 102631. 31981982 10.1016/j.ebiom.2020.102631 PMC6992943

[phy215813-bib-0052] Warden, C. H. , & Fisler, J. S. (2008). Comparisons of diets used in animal models of high‐fat feeding. Cell Metabolism, 7(4), 277. 18396128 10.1016/j.cmet.2008.03.014 PMC2394560

[phy215813-bib-0053] Wculek, S. K. , & Malanchi, I. (2015). Neutrophils support lung colonization of metastasis‐initiating breast cancer cells. Nature, 528(7582), 413–417. 26649828 10.1038/nature16140 PMC4700594

